# Insights into the dynamic cell associated and secreted proteome of *Staphylococcus aureus* cultured in TSB and milk media: a proteomic analysis

**DOI:** 10.1186/s12866-025-04587-z

**Published:** 2025-12-27

**Authors:** Wanting Zhu, Yujing Wang, Yanxin Li, Changjiang Zang, Hongning Jiang, Qijing Du, Jun Wang, Rongbo Fan, Rongwei Han, Yongxin Yang

**Affiliations:** 1 College of Food Science and Engineering, Qingdao Agricultural University, Qingdao, Shandong 266109 China; 2 College of Animal Science, Xinjiang Agricultural University, Urumqi, Xinjiang 830052 China

**Keywords:** *Staphylococcus aureus*, Quantitative proteomics, Cell associated proteins, Secretory proteins

## Abstract

The availability of nutrients is closely linked to the protein expression profile of microorganisms. In this study, a comparative investigation has been performed to examine the expression patterns of cell associated proteins and secreted proteins in *Staphylococcus aureus* cultivated in TSB and milk media at three time points: 3 h, 9 h, and 18 h of the growth stage using proteomic techniques. In TSB medium, cell-associated proteins such as acetyl-CoA acyltransferase, acyl-CoA synthetase, argininosuccinate lyase, and argininosuccinate synthase were detected at higher levels at 9 h and 18 h compared with 3 h, indicating overall increases during growth. While in milk, quinone oxidoreductase 1 and zinc-type alcohol dehydrogenase-like protein SAR2277 exhibited a significant increase across 3, 9 and 18 h. Secretory proteins such as δ-hemolysin, phospholipase C and panton-valentine leukocidin F showed significant increases in both media across 3, 9 and 18 h. Notably, cell associated proteins or secreted proteins such as the leukocidin S subunit and staphopain B were significantly upregulated in TSB medium, while thermonuclease and transcriptional regulator SarA were found to increase in milk. These findings highlight the importance of the culture medium in determining the relative abundances of cell associated proteins and secretory proteins in *S. aureus*, providing insight into microbial adaptation mechanisms. Furthermore, these up-regulated proteins may serve as potential biomarkers and have practical implications for improving control strategies, leading targeted approaches to reducing *S. aureus* contamination in milk and dairy products, and eventually contributing to better food safety and quality.

**Supplementary Information:**

The online version contains supplementary material available at 10.1186/s12866-025-04587-z.

## Introduction

*Staphylococcus aureus* (*S. aureus*) is a common pathogenic bacterium frequently detected in milk [1, 2], with detection rates varying across different geographical regions and seasons [3, 4]. This bacterium plays a crucial role in altering milk composition through the enzymatic breakdown of casein, thereby impacting the quality and nutritional value of the milk [5, 6]. The ability of *S. aureus* to proliferate in dairy products and produce a range of harmful toxins, which pose significant health risks to consumers, has been well documented [7]. The enterotoxins produced by *S. aureus* exhibits significant heat resistance, such that it may survive even after high-temperature pasteurization of dairy products [8, 9]. Consequently, monitoring *S. aureus* levels in milk is essential for ensuring food safety and safeguarding public health.

In recent years, significant advances have been made in the research of *S. aureus*, encompassing its pathogenic mechanisms, drug resistance, and other related aspects [10–12]. There is also considerable interest in understanding how growth media and environmental variables influence protein expression in *S. aureus.* Different growth conditions have been demonstrated to influence the expression of a wide range of proteins, including those critical for metabolism, pathogenicity, and stress responses [13]. For instance, research into the effects of growth media composition on *S. aureus* has shown that cultivation in Dulbecco’s Modified Eagle Medium or Tryptic Soy Broth (TSB) markedly affects the bacterium’s capacity for biofilm formation [14]. At the transcriptional level, a comparison of cation-adjusted Mueller-Hinton broth and Roswell Park Memorial Institute 1640 Medium supplemented with 10% Luria broth revealed differential expression in over 800 genes [15]. At the protein level, a total of 60 differentially regulated cytoplasmic proteins were detected in *S. aureus* grown in TSB medium supplemented with different concentrations of NaCl, and alterations in proteins associated with protein synthesis were higher in cells grown with NaCl compared to those grown without salt [16].

The proteins of *S. aureus* have been categorized into several groups, including cytosolic proteins, membrane-bound proteins, cell surface-associated proteins, and extracellular proteins [17, 18]. Chronic infections, primarily associated with *S. aureus* extracellular proteins, persist due to the pathogen’s ability to evade host immune responses through the secretion of various virulence factors [19]. Secreted toxins, such as exotoxins, account for about 10% of the total secretome (around 1354 proteins) [20, 21] and confirmed the important role of extracellular virulence factors in the pathogenesis of *S. aureus* [22]. The expression of these virulence factors in *S. aureus* is recognized to fluctuate based on the growth environment [23]. For instance, triacylglycerol 1 and 2 were secreted by *S. aureus* in a NaCl-supplemented medium [16]. The capacity of *S. aureus* to thrive in milk is intricately linked to its pathogenicity, with enterotoxins in particular being secreted into the milk during bacterial proliferation [24, 25]. It was illustrated that although *S. aureus* strains produce lower levels of the staphylococcal enterotoxin D and staphylococcal enterotoxin R in milk compared to microbial broth, some strains are still capable of secreting over 2 µg/mL of staphylococcal enterotoxin D in milk [26]. As previously reported, a few studies investigated the dynamic changes in the microbial proteome and secretome of *S. aureus* cultured in different media [22, 27].

The aim of this study is to utilize a label-free quantitative proteomics approach to systematically investigate the differential expression of cell associated proteins and secreted proteins in *S. aureus* cultured in milk and TSB media at different growth stages. The results of this study may offer novel insights into the interplay between protein expression and nutritional factors, thereby contributing to the improvement of milk safety standards and quality control measures.

## Materials and methods

### Determination of bacterial growth curve

TSB medium (Qingdao Haibo Biotechnology Co., Ltd.), composed of 17.0 g casein tryptone, 5.0 g NaCl, 3.0 g soybean papain hydrolysate, 2.5 g di-potassium hydrogen phosphate and 2.5 g glucose, was prepared by dissolving the components in MilliQ water at a concentration of 3.0% (w/v). Skim milk powder was purchased from Inner Mongolia Yili Industrial Group, the milk powder samples were re-dissolved with MilliQ water at a ratio of 1:9 (W/V) under stirring at 40 °C for 1 h. The growth curve of *S. aureus* (ATCC 25923) was assessed in both TSB and milk media at 37 °C with shaking at 225 rpm/min. The milk culture was counted by plate colony counting method every hour from 0 to 10 h and every 3 h from 12 to 24 h to finalize the bacterial growth curve in milk. The growth of *S. aureus* in the TSB medium was monitored with 600 nm optical density values during the same period.

As illustrated in Fig. S1, the growth period of *S. aureus* was divided into three parts, 1–3 h for the log phase, 3–9 h for the logarithmic period, and 9–18 h for the stationary phase. Samples were taken at 3 h, 9 h, and 18 h, respectively, representing the growth of *S. aureus* at the transition period between phases.

### Bacterial isolation

Samples were collected at 3, 9, and 18 h from *S. aureus* cultures grown in TSB medium. The culture was transferred to a sterile 50 mL conical centrifuge tube and centrifuged at 9,000 × g for 10 min at 4 °C to collect the bacterial cells, then washed three times with phosphate-buffered saline, followed by rapid freezing to minimize cell lysis and protein degradation. As well, the supernatant was collected for secreted proteins.

For *S. aureus* cultured in milk, the cultured milk was transferred to a sterile 50 mL conical centrifuge tube and centrifuged at 9,000 × g for 10 min at 4 °C to collect the bacterial cells and casein micelles, while the supernatant was retained for the extraction of secreted proteins. The cell pellet was washed 3 times with phosphate-buffered saline, then washed 3 times with 500 mM EDTA solution to dissociate from the casein micelles until the washings were clarified and centrifuged at 4 °C to retain the cells pellet. The resulting pellet was subsequently stored at −20 °C until further use.

### Extraction of cell associated protein

The bacterial cells were ground in a sterile mortar with liquid nitrogen and subsequently resuspended in the SDT lysis buffer, which consists of 4% SDS (W/V), 100 mM dithiothreitol, and 100 mM Tris-HCl buffer (pH 7.6). The lysis buffer cell suspension was sonicated on ice at 200 W for 10 min (8 s on, 5 s off) using an ultrasonic cell disruptor (ATPIO XO-1000D, China). After sonication, the mixture was centrifuged at 10,000 × g for 10 min. The supernatant was collected and subjected to protein precipitation by the addition of four volumes of pre-cooled acetone, followed by incubation at −20 °C overnight. The protein pellet was obtained by centrifugation at 10,000 × g for 10 min and subsequently washed twice with pre-cooled acetone. The resulting pellet was then resuspended in 100 mM Tris-HCl buffer, and the protein concentration was determined using the Bicinchoninic Acid Assay protein assay kit (Thermo Fisher Scientific, USA).

### Extraction of secreted proteins

The TSB media or milk supernatant from the isolated bacteria grown after 3 h, 9 h, and 18 h was collected respectively and mixed with an equal volume of trichloroacetic acid solution (20% w/v) to induce protein precipitation. The resulting protein precipitate was washed twice with pre-cooled acetone and subsequently reconstituted in 100 mM Tris-HCl buffer. The protein concentration was then determined as above-mentioned.

### Protein digestion

Thirty micrograms of bacterial cell associated proteins and secreted proteins from each sample of species were mixed with 4% SDS and 30 mM Tris-HCl, pH 7.6, and reduced in a 100 mM dithiothreitol solution at 65 °C for 45 min. After the samples cooled, the protein solution was mixed with 200 µL UT buffer (8 M urea, 100 mM Tris-HCl, pH 8.5), applied to a 10-kDa cut-off filter tube (Sartorius, Göttingen, Germany), and centrifuged at 14,000 × g for 25 min. After washing the samples with UT buffer, alkylation was performed using 50 mM iodoacetamide in the dark at 25 °C for 45 min. The protein samples were then digested with 100 µL of trypsin solution (1 µg sequencing-grade trypsin in 50 mM ammonium bicarbonate) in a water bath at 37 °C for 16–18 h. The digestion reaction was terminated. by adding 10 µL of formic acid. Next, the peptide mixture was loaded onto a C18 column (60108–303; Thermo Fisher Scientific, MA, USA) for desalting. The samples were dried in a speed vacuum and placed at − 80 °C. Three independent biological replicates were conducted for each experimental condition.

### Data-dependent acquisition analysis by LC-MS/MS

The peptide mixtures were dissolved in 0.1% formic acid and analyzed using Easy nLC 1000 chromatography coupled with an Orbitrap Fusion Lumos mass spectrometer (Thermo Fisher Scientific, CA, USA). Digested peptides were initially loaded onto a C18 trap column (100 μm × 2 cm, 5 μm; Thermo Fisher Scientific) using solution A (0.1% formic acid) at a flow rate of 300 nL/min, and subsequently separated on a C18 analytical column (100 mm × 75 μm, 3 μm; Thermo Fisher Scientific). The separation gradient was programmed with solution B (0.1% formic acid in 80% acetonitrile) as follows: 0–10% B over 3 min, 10–35% B over 45 min, 35–80% B over 26 min, ramping to 100% B within 1 min, and maintaining 100% B for an additional 15 min.

For proteomic analysis, the Orbitrap Fusion Lumos MS operated in positive ion mode with a parent ion scan range of 300–1800 m/z. Automatic switching between MS and MS/MS modes was enabled. The survey scan parameters were set to a resolution of 60,000, automatic gain control (AGC) target of 400,000, maximum injection time of 50 ms, and exclusion duration of 40 s. The top 20 most intense precursor ions with a charge ≥ 2 from the survey scan were selected for higher-energy collisional dissociation fragmentation. The MS/MS parameters included normalized collision energy of 27 eV, MS/MS resolution of 15,000, AGC target of 50,000, and maximum injection time of 50 ms.

### Protein identification and quantification

The raw files were processed through MaxQuant software (version 2.0.3.0) to search against data downloaded from the UniProtKB database (*S.aureus* 11217 entries; downloaded December 2023) [28]. The parameters were set as follows: trypsin/P was designated as the digestion enzyme with a maximum allowance of two missed cleavage sites. Carbamidomethylation of cysteine residues was specified as a fixed modification, while N-terminal acetylation and methionine oxidation were defined as variable modifications. Matching between runs was enabled with a retention time tolerance of 0.7 min and an ion mobility tolerance of 0.05. Protein quantification was carried out using a label-free quantification (LFQ) approach, based on the intensities of razor and unique peptides. Protein and peptide identifications were conducted with a false discovery rate and peptide-spectrum match threshold set at 0.01.

### Quantitative real-time PCR (RT-qPCR) analysis

The total RNA of *S. aureus* grown in TSB and milk at different stages was extracted from cells using a Bacteria Total RNA Isolation Kit (Sangon Biotech Co., Ltd., Shanghai, China). Total RNA was reverse transcribed and amplified using the BeyoFast™ SYBR Green One-Step qRT-PCR Kit (Beyotime, China). Four differential abundant proteins were selected and their mRNA and *gyrB* expression levels were determined using RT-qPCR, each gene was performed in triplicate (Table S1). Statistical analysis was conducted using SPSS (Statistical Package for the Social Sciences).

### Bioinformatics and statistical analysis

Protein identification was accepted if the protein was detected in two out of three biological replicates, with each detection supported by at least two peptides, and these proteins were used for subsequent data analysis. The quantified proteins were subjected to Perseus software (www.maxquant.org/perseus/) for statistical analysis and principal component analyses (PCA). One-way analysis of variance (ANOVA) was conducted to evaluate differences in protein abundance among groups. Differentially abundant proteins were identified based on a |log_2_ (fold change) | ≥ 1 and a *P*-value < 0.05. These differential abundant proteins were subjected to TBtools-II (Toolbox for Biologists) v2.083 software for gene ontology enrichment and Kyoto Encyclopedia of Genes and Genomes (KEGG) pathways. The data of selected genes obtained by RT-qPCR were analyzed using one-way ANOVA followed by Duncan’s test in IBM SPSS software. *P*-value < 0.05 was defined as statistical significance.

## Results

### Identification and analysis of cell associated proteins

Based on the label free proteomics approach, a total of 14,490 peptides were identified from cell associated proteins of *S. aureus* cultured in TSB medium, whereas 12,259 peptides corresponding to 909 distinct proteins were detected from cell associated proteins of *S. aureus* grown in milk (Fig. S2). The identified cell associated proteins from both milk and TSB media were subjected to PCA. As shown in Fig. S3, the PCA score plots of cell-associated proteins in *S. aureus* revealed a clear separation between the two media, whereas the secreted proteins from *S. aureus* cultivated in milk and TSB exhibited similar expression profiles across the three growth phases (3 h, 9 h, and 18 h). These findings indicate that the composition of the culture medium exerts a pronounced influence on the overall expression patterns of cell-associated proteins in *S. aureus*. In this study, the selection of differentially expressed proteins was primarily based on their significant changes in abundance, with screening criteria of |log₂ (fold change) | ≥ 1 and *P* < 0.05.

#### *S. aureus* cell associated proteins in TSB medium

Statistical analysis of *S. aureus* cell associated proteins in TSB medium indicated that several proteins followed unique temporal patterns among the 3, 9, and 18 h samples. Acetyl-CoA acyltransferase (FadA), acyl-CoA synthetase (FadD), argininosuccinate lyase, argininosuccinate synthase, formate acetyltransferase, and NAD-specific glutamate dehydrogenase showed an increase in abundance across 3 h, 9 h, and 18 h. The abundance of aldehyde-alcohol dehydrogenase, N-acetylmuramoyl-L-alanine amidase sle1 (Sle1), multicopper oxidase (Mco), and pyruvate kinase increased between 3 and 9 h, then decreased between 9 and 18 h growth. Moreover, the abundances of translation initiation factor IF-1, adapter protein MecA, tetratricopeptide repeat protein, 30 S ribosomal protein S18, and fructose-1,6-bisphosphate aldolase decreased between 3 and 18 h.

#### *S. aureus* cell associated proteins in milk medium

In milk medium, the abundance of several proteins, including quinone oxidoreductase 1 (Mqo1), carbamate kinase 1, and zinc-type alcohol dehydrogenase-like protein SAR2277 exhibited a significant and consistent increase from 3 to 18 h. The abundance of transketolase increased between 3 and 9 h but decreased between 9 and 18 h. Conversely, the abundance of DHHA1 domain protein and proline/betaine transporter decreased between 3 and 9 h and then increased between 9 and 18 h. Meanwhile, the protein abundance of cold shock protein CspA, cell-division initiation protein and delta-hemolysin decreased between 3 and 18 h.

#### Comparative analysis of *S. aureus* cell associated proteins in TSB and milk media

Regarding the differences in *S. aureus* cell associated proteins between media, the levels of thiol peroxidase, 4-hydroxy-tetrahydrodipicolinate synthase, and tyrosine–tRNA ligase were higher in TSB medium than in milk between 9 and 18 h, suggesting more active cellular metabolism under nutrient-rich TSB conditions. However, several proteins, such as acyl carrier protein, Sle1, and phosphatidylglycerol–prolipoprotein diacylglyceryl transferase were greater in milk than in TSB medium between 9 and 18 h, indicating that *S. aureus* adapts to the milk environment by enhancing its cell envelope integrity and modifying its surface structures to cope with environmental stress and nutrient limitation.

**Fig. 1:**
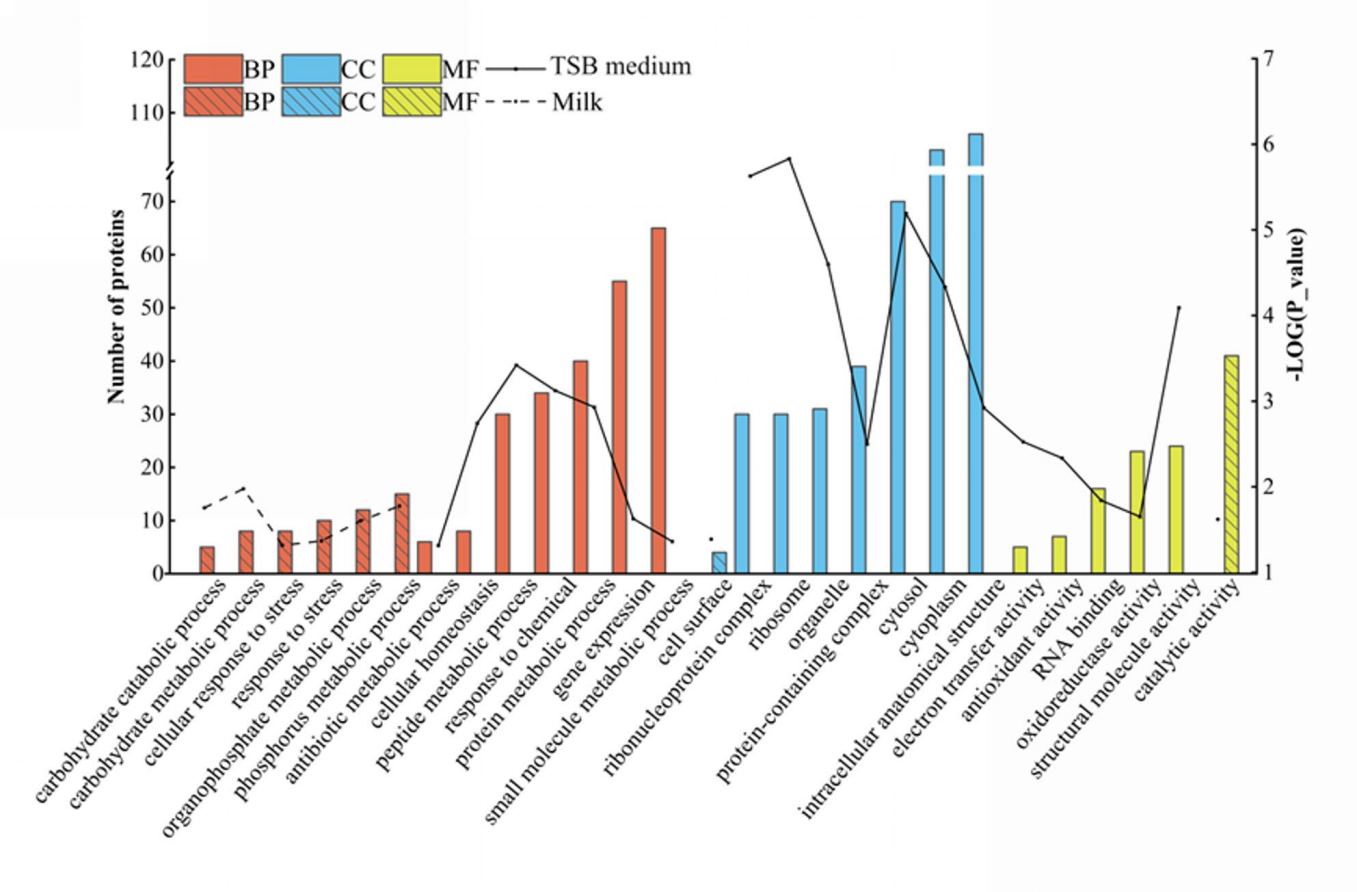
Gene ontology analysis of differentially abundant cell associated proteins of *S. aureus* cultured in TSB and milk media. The solid and dashed lines represent − log(*P*-value) of functional enrichment significance in TSB medium and milk, respectively. BP, biological process; CC, cellular component; MF, molecular function

Protein annotations were used to classify differentially abundant *S. aureus* cell associated proteins into biological processes, cellular components, and molecular functions. At the biological process level, the most abundant proteins in TSB medium were primarily associated with response to chemical, cellular homeostasis, small molecule metabolic process, protein metabolic process, and gene expression. In contrast, in milk, the predominant processes included response to stress, phosphorus metabolic process, carbohydrate metabolic process, organophosphate metabolic process, and carbohydrate catabolic process. At the cellular component level, in TSB, the majority of proteins were associated with the protein-containing complex, ribonucleoprotein complex, cytosol, organelle, cytoplasm, intracellular anatomical structure, and ribosome, whereas in milk, proteins were primarily involved in the cell surface. Regarding molecular functions, in TSB, the most common functions were structural molecule activity, electron transfer activity, antioxidant activity, RNA binding, and oxidoreductase activity, while in milk, catalytic activity was more prominent (Fig. 1).

The KEGG pathway analysis of differentially abundant proteins in *S. aureus* cultivated in TSB medium and milk is summarized in Table S2. The differential abundance of cell associated proteins under these two growth conditions was associated with a variety of pathways, including carbohydrate metabolism, translation, energy metabolism, pyruvate metabolism, glycolysis/gluconeogenesis, chaperones and folding catalysts. Notably, specific pathways such as amino acid metabolism, nucleotide metabolism, purine metabolism, the citric acid cycle, and xenobiotics biodegradation and metabolism were predominantly identified in the TSB medium. In contrast, the milk medium showed strong associations with pathways including transfer RNA biogenesis, exosome, mitochondrial biogenesis, aminoacyl-tRNA biosynthesis, oxidative phosphorylation and carbon fixation in photosynthetic organisms.

### Identification and analysis of secreted proteins

#### Secreted proteins of *S. aureus* in TSB medium

In TSB medium, a total of 6,985 peptides corresponding to 552 secreted *S. aureus* proteins were identified, whereas 4,605 peptides corresponding to 480 secreted proteins were detected during bacterial growth in milk (Fig. S4). We quantified secreted proteins from both milk and TSB media. These proteins were subjected to PCA analysis. The PCA score plots showed distinct clusters for each of the three stages: 3 h, 9 h, and 18 h. This clustering occurred in both media. The results indicated a clear separation of protein profiles associated with proteins released at different stages in TSB and milk media (Fig. S3).

The relative abundances of these proteins varied significantly over time. In TSB medium, peptidase T (PepT), staphopain B, phospholipase C (Hlb), triacylglycerol lipase, γ-hemolysin component B (HlgB), delta-hemolysin (Hld), bifunctional autolysin, and panton-Valentine leukocidin F (LukF-PV) demonstrated an increase in abundance from 3 to 18 h. Moreover, the levels of superoxide dismutase [Mn/Fe] (SodM), type VII secretion system extracellular protein A (EsxA), and clumping factor A increased significantly between 3 and 9 h, then decreased between 9 and 18 h. The protein abundance of bacilliredoxin SAS1371, UDP-N-acetylglucosamine 1-carboxyvinyltransferase 1, iron-sulfur cluster carrier protein, and uridylate kinase decreased between 3 and 18 h.

#### Secreted proteins of *S. aureus* in milk medium

In the milk medium, the secreted proteins of phospholipase C, Hlb, HlgB, Hld, transcriptional regulator SarA (SarA), glyoxal reductase, and LukF-PV increased abundance in milk across 3, 9, and 18 h. The abundance of thermonuclease (Nuc) was observed to increase significantly between 3 and 9 h, and then remain unchanged between 9 and 18 h. In addition, the abundance of ornithine carbamoyltransferase, iron-sulfur cluster repair protein ScdA, and EsxA increased between 3 and 9 h and decreased between 9 and 18 h. Furthermore, the abundance of SodM, 30 S ribosomal protein S19, and iron-sulfur cluster carrier protein decreased between 3 and 18 h.

#### Comparative analysis of secreted proteins of *S. aureus* in TSB and milk media

Throughout the growth stages, the abundance of several proteins, including urease subunit alpha (UreC), Hlb, LukF-PV, HlgB, and Hld, increased significantly across 3, 9, and 18 h in both TSB and milk media. In addition, the levels of PepT, staphopain A, and cell division protein SepF were greater in TSB medium than in milk at 9 h and 18 h, while several proteins, such as oligoendopeptidase F, SarA, and serine-aspartate repeat-containing protein D were higher in milk than in TSB medium.

**Fig. 2:**
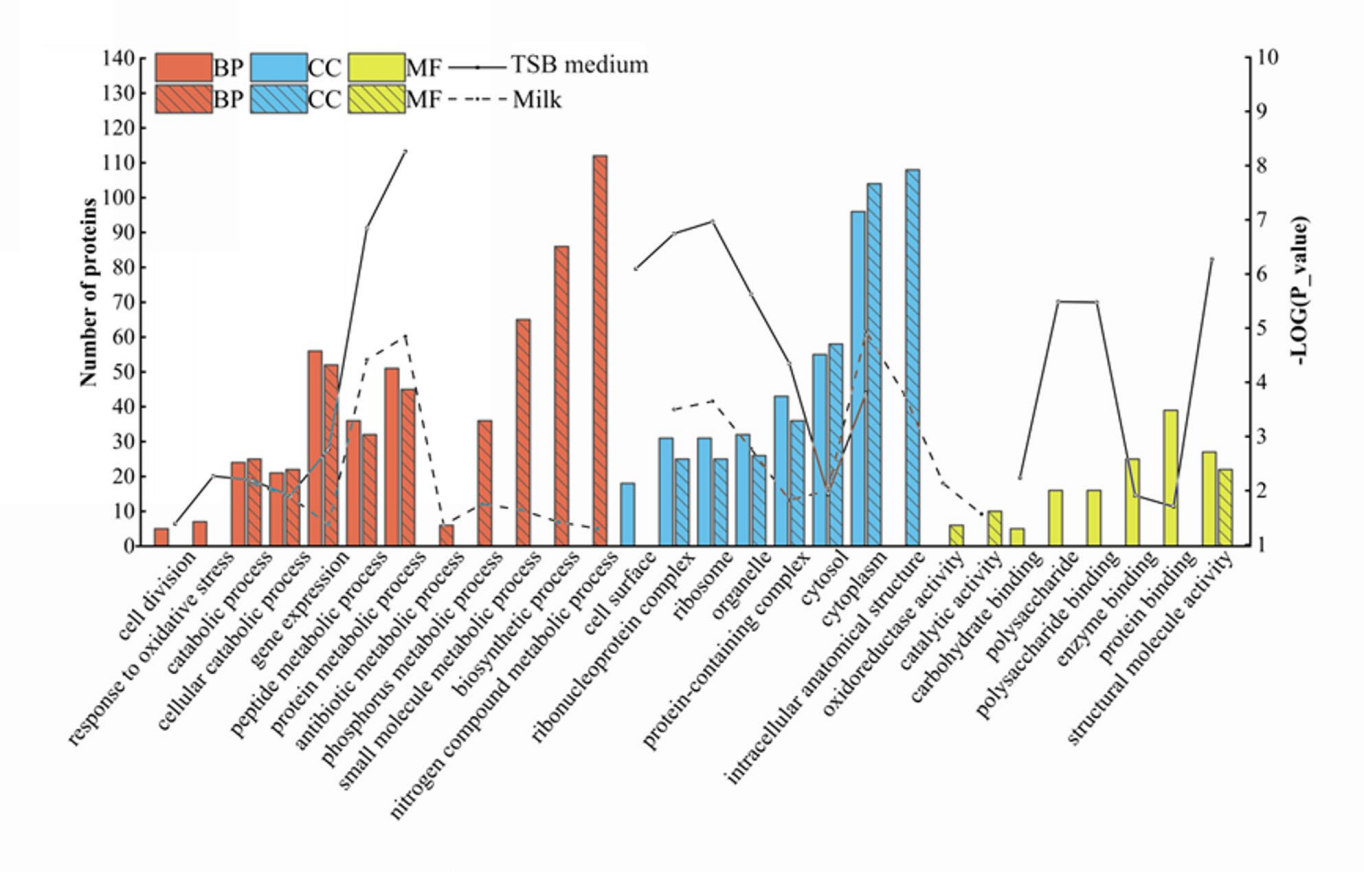
Gene ontology analysis of differentially abundant secreted proteins of *S. aureus* cultured in TSB and milk media. The solid and dashed lines represent the − log(*P*-value) of functional enrichment significance in TSB medium and milk, respectively. BP, biological processes; CC, cellular components; MF, molecular functions

Protein annotations revealed that the differentially abundant secreted proteins of *S. aureus* in both TSB and milk were mainly associated with the functions such as metabolic and catabolic process, cytosol and cytoplasm components, structural molecule activity (Fig. 2).

Table S3 summarizes the KEGG pathway analysis of differentially abundant secreted proteins of *S. aureus* in TSB and milk media. Overall, these proteins were associated with carbohydrate metabolism, translation, amino acid metabolism, energy metabolism, ribosome, pyruvate metabolism, exosome, membrane trafficking, glyoxylate and dicarboxylate metabolism pathways. Notably, several pathways were uniquely enriched in TSB, including pyrimidine metabolism, bacterial toxins, translation factors, lipid biosynthesis, nicotinate and nicotinamide metabolism, RNA polymerase and transcription. In contrast, pathways related to lipid metabolism, xenobiotic biodegradation and metabolism, nucleotide metabolism, the pentose phosphate pathway, and benzoate degradation were predominantly enriched in the milk medium.

### Supporting the proteomic data at the mRNA level

**Fig. 3:**
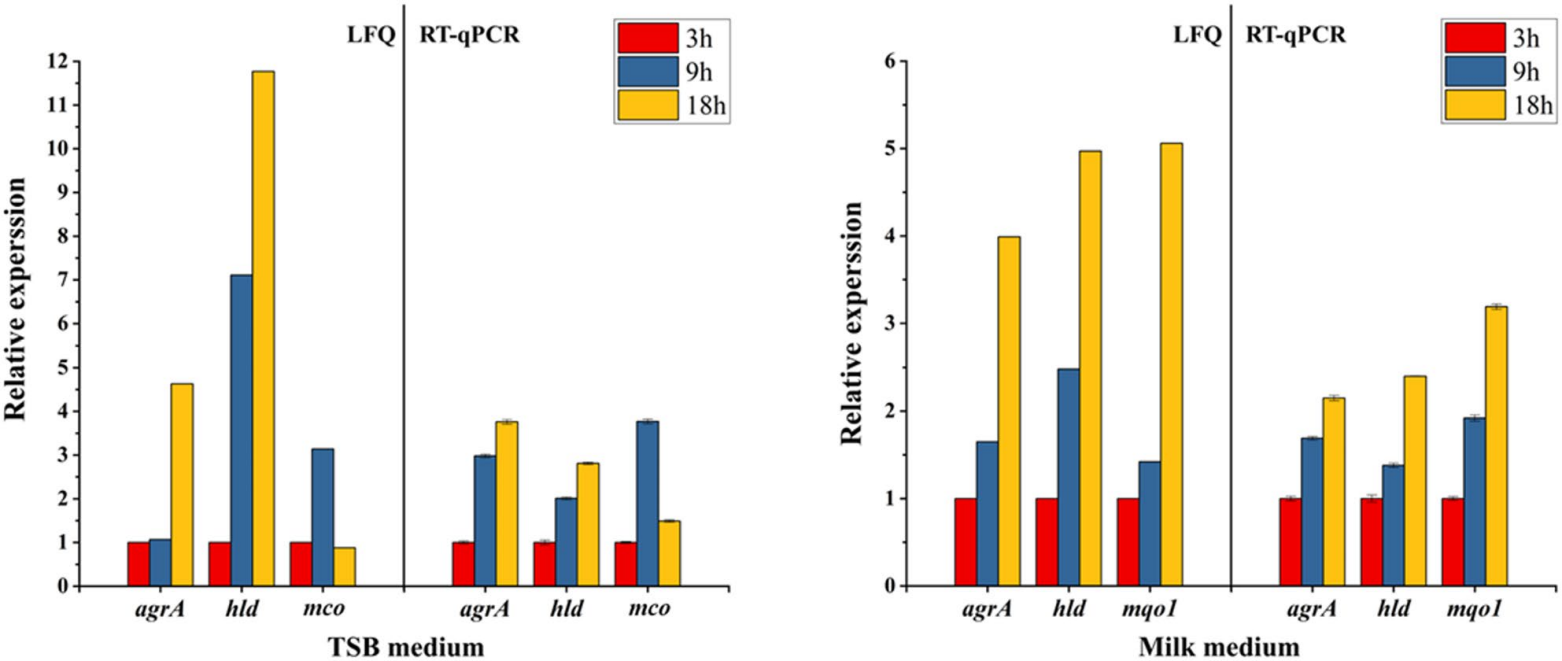
RT-qPCR analysis of genes corresponding to proteins identified in the proteomic analysis of *S. aureus* grown in either milk or TSB media

To support and complement the proteome data obtained through LFQ analysis, the expression patterns of selected proteins and their corresponding genes were analyzed using quantitative reverse transcription polymerase chain reaction (RT-qPCR). The results showed a time-dependent increase in the expression of the *AgrA* and *Hld* genes in TSB medium across 3, 9 and 18 h. Furthermore, *mco* expression increased between 3 and 9 h and subsequently declined between 9 and 18 h in the TSB medium. In contrast, *ureC*, *hld*, and *mqo1* expression rose consistently in the milk medium over time (Fig. 3). These expression profiles were relatively consistent with the LFQ proteomic results, showing a link between protein abundance variations and gene expression levels.

## Discussion

### Changes in cell associated proteins of *S. aureus* both conditions

The protein abundances of FadA and FadD increased in TSB medium across 3, 9 and 18 h. Consistent with previous studies using microarray analysis, the *FadA* and *FadD* genes were highly elevated within two days of cultivation [29]. Specifically, FadA was responsible for conjugating long-chain fatty acids with coenzyme A to produce fatty acyl-CoA, which played a critical role in lipid synthesis and repair by facilitating the transport of fatty acyl groups [30]. FadD, on the other hand, catalyzes the conversion of free fatty acids into fatty acyl-CoA by binding with Coenzyme A (CoA), a process known as fatty acid “activation”, which is the first step in fatty acid metabolism. Fatty acyl-CoA is the active form of fatty acids, enabling their further metabolism through various pathways, such as β-oxidation (which breaks down fatty acids into acetyl-CoA) and fatty acid synthesis [31]. Moreover, the increased expression of FadA and FadD indicated that *S. aureus* was actively engaging in fatty acid metabolism to adapt to the nutritional circumstances in TSB medium, perhaps boosting its adaptability.

In TSB medium, the abundance of Mco was significantly higher at 9 h compared to 3 h, followed by a notable decrease at 18 h. Previous research used TSB medium to culture *S. aureus* and quantitatively analyze Mco transcript levels using RT-qPCR, indicating a considerable rise in Mco expression during the growth phase [32]. The elevated Mco expression during the pre-growth phase could be associated with oxidative stress [33]. Mco, an important antioxidant enzyme in bacteria, is involved in the oxidative stress pathway, where redox equilibrium within cells is effectively maintained through the catalysis of superoxide dismutation and the regulation of the oxidation states of metal ions, thereby mitigating their toxicity [34]. In addition, Mco is involved in the copper homeostasis pathway, where bacteria acclimate to growth conditions by maintaining internal copper levels at the lowest concentration required for growth, which results in a decrease in abundance of Mco proteins [35].

The abundance of Mqo1 increased significantly in milk across 3, 9 and 18 h. Quantitative qRT-PCR analysis demonstrated a marked upregulation of *mqo1* expression over a 24-hour period in TSB supplemented with 0.5% glucose [36]. Mqo1, an enzyme engaged in the TCA cycle, was responsible for oxidizing malate to oxaloacetate, which is necessary for gluconeogenesis [37]. Notably, the chemical composition of TSB medium contains a higher concentration of glucose compared to milk. The significant increase of Mqo1 expression in milk may be attributable to the lack of immediately available glucose for *S. aureus* in milk, requiring the bacterium to metabolize alternative substrates to create energy for growth and reproduction. The cell-associated differential protein such as Mqo1 may serve as potential biomarkers for evaluating milk quality and early monitoring of *S. aureus* infected with dairy cows.

### Changes in secreted proteins of *S. aureus* under different growth conditions

Both TSB and milk are nutritionally rich environments that support the vigorous growth of *S. aureus*. Accordingly, secreted proteins associated with biological processes and cellular components were markedly upregulated in both media, reflecting the metabolically active state of *S. aureus* under nutrient-abundant conditions. The abundance of UreC showed a significant increase across 3, 9 and 18 h in both milk and TSB media. Furthermore, UreC expression in *S. aureus* was dramatically enhanced during the exponential growth phase when cultivated in Chemically Defined Medium, as shown by proteomic studies [38]. UreC is an essential enzyme in the urea metabolism pathway, catalyzing the hydrolysis of urea to yield ammonia and carbon dioxide. This process helps to regulate the pH within cells and promote energy metabolism through acetyl-CoA synthetase and the tricarboxylic acid cycle, providing the essential energy for cell development and adaptability [39]. The enzymatic activity of UreC was critical for maintaining the energy balance needed for cell development and adaptability.

A significant increase in the abundance of hemolysin-related Hld, Hlb and HlgB was observed between 3 and 18 h in both milk and TSB media, aligning with a high expression of *Hld* mRNA in *S. aureus* during both the growth and stationary phases cultured in LB medium [40]. Most *S. aureus* isolates produce hemolysin, which is generally recognized as a key element in the pathogenicity of infections caused by these bacteria [41, 42]. Hemolysin was recognized to be selectively interacting with the phospholipid bilayer of host cell membranes, being introduced into the membrane and producing modifications in its structure and permeability. This process causes the creation of ion channels or pores, which facilitates the passage of ions and small molecules, disturbing the internal and exterior cellular environments and eventually leading to cell lysis [43, 44]. These disturbances played an important role in the infection process, facilitating bacterial entry and survival within the host. The abundance of LukF-PV was significantly elevated in both milk and TSB media across 3, 9 and 18 h; however, its expression levels did not differ significantly between the two conditions at corresponding time point. In a previous study, *LukF-PV* was considerably elevated during the growth phase of *S. aureus* cultured in BHI medium using quantitative RT-PCR [45]. LukF-PV has been shown to bind specifically to host leukocytes, where it facilitates the formation of transmembrane pores or channels in the cell membrane. The resulting disruption of ionic and molecular homeostasis across the membrane ultimately leads to leukolysis [46]. Furthermore, LukF-PV was found to induce macrophage death, mediate the escape of *S. aureus* from the host cell, thereby facilitating the survival of *S. aureus* [47]. Thus, an increased abundance of LukF-PV augments *S. aureus* virulence by targeting and lysing host immune cells, thereby facilitating bacterial survival and persistence. Interestingly, the expression of LukF-PV is regulated by several transcriptional regulators, including two-component systems, quorum sensing mechanisms, and the accessory gene regulator (*Agr*) system [48]. In the present study, despite the elevated expression of Agr in milk, comparable levels of LukF-PV were observed in both milk and TSB media. The similar expression of LukF-PV across these two distinct environments suggests that the bacterial regulatory mechanisms governing toxin production in *S. aureus* are resilient and effectively operational in diverse conditions. This finding suggests that LukF-PV production in *S. aureus* is governed by multifactorial regulatory mechanisms that enable toxin synthesis under diverse environmental conditions, underscoring its potential role in the pathogenicity of *S. aureus*. However, further investigations are required to elucidate the precise regulatory mechanisms involved.

Nevertheless, the compositional disparities between TSB and milk created distinct metabolic interactions between the microbes and their environment. SodM secretion in TSB medium increased significantly between 3 and 9 h, then decreased between 9 and 18 h, whereas it decreased in milk across 3, 9 and 18 h. The RT-qPCR study revealed a considerable increase in *SodM* expression over 24 h of *S. aureus* growth in TSB [49]. *S. aureus* contains two superoxide dismutases, SodA and SodM, with SodM playing an important role in mitigating the detrimental effects of toxic superoxide anion radicals produced within the bacteria [50]. SodM, an enzyme distinct to *S. aureus*, catalyzes the dissociation of superoxide anion radicals into molecular oxygen and hydrogen peroxide [51], thereby reduces oxidative stress damage to bacteria, maintains intracellular redox balance, and preserves their normal physiological state [52]. Although these radicals are produced within the bacterial cytoplasm, their generation and the associated oxidative stress can be modulated by the extracellular environment. The decline of SodM of *S. aureus* in milk could be explained by the low concentration of superoxide anion radicals present in this medium. Antioxidant components found in milk, such as vitamin C and E compounds, successfully scavenged these free radicals, eliminating the need for SodM activity in this environment [53]. Additionally, PepT is more abundant in TSB than in milk medium, where it primarily catalyzes the removal of the N-terminal amino acid from most tripeptides. PepT1 and PepT2 have been shown to be non-essential for nutrient acquisition in peptide-rich media; however, their expression is regulated by the global virulence factor regulator SarA, implicating them as key contributors to the virulence of several bacterial pathogens [54]. Furthermore, pathways associated with bacterial toxins, human diseases, and *S. aureus* infection were also enriched in TSB, indicated by active protein synthesis and metabolic processes that promote the production of virulence factors. These further support the enhanced expression of virulence factors, such as hemolysins and lipases, which are pivotal in the pathogenicity of *S. aureus* [55]. These findings collectively underscore the dynamic nature of *S. aureus* metabolism, emphasizing how nutrient-rich environments like TSB enhance its pathogenic potential. The analysis provides valuable insights into how *S. aureus* adapts to its surroundings, optimizing the expression of virulence factors that are critical for its ability to cause infections.

The relative abundances of Nuc increased significantly between 3 and 9 h, then it remained unchanged between 9 and 18 h in milk. *S. aureus* cultivated in BHI broth showed an observable increase of *Nuc* expression during the mid-logarithmic growth phase, which was confirmed by quantitative RT-PCR [56]. The staphylococcal nuclease, which is controlled by the *Nuc* gene, acted as a thermostable nucleic acid enzyme capable of hydrolyzing DNA and RNA within host cells, hence contributing to tissue damage and increasing the pathogenicity of *S. aureus* by facilitating its spread and colonization [57]. Moreover, the ability of the nuclease to break down host nucleic acids served as a crucial nutrient source for *S. aureus*, with the released nucleotides and nucleic acids from host tissues being ingested by the bacterium to maintain their growth and survival within the host environment [58]. Specific growth conditions altered the regulation of Nuc expression, with the gene being upregulated during distinct growth phases, particularly in environments with limited nutrients or under conditions that mirrored the host’s physiological state [59]. Conversely, in the nutrient-rich environment of TSB, Nuc expression may have been repressed because the bacteria could receive sufficient nutrients without breaking down host nucleic acids. This suggests that the regulation of Nuc expression is linked to the availability of external resources, with nutrient-rich conditions reducing the necessity for the bacterium to engage in host tissue degradation for nutritional acquisition. Additionally, the enrichment of pathways related to lipid metabolism, xenobiotic biodegradation, and nucleotide metabolism indicates metabolic reprogramming that enables *S. aureus* to efficiently utilize milk-derived substrates such as fatty acids and nucleotides. However, the molecular mechanisms underlying the ability of *S. aureus* to adapt to nutrient availability in the milk environment and to modulate protein biosynthesis, thereby promoting its persistence and pathogenic potential, require further investigation using targeted and integrative omics approaches. The secreted proteins such as Hld, LukF-PV, and Nuc, which are bacterial toxins produced by *S. aureus*, deserve particular attention in the dairy industry, as they could help assess the hygienic status and *S. aureus* infection. Such biomarkers may provide valuable targets for early detection and management strategies aimed at preventing contamination and controlling the infection of *S. aureus*, as well as for evaluating the risk of toxin-associated foodborne illnesses. Nevertheless, further experimental validation is required to assess the potential health risks of these proteins to consumers.

The observed proteomic alterations likely represent the outcome of complex interactions between *S. aureus* and its surrounding culture environment. The increased expression of acyl-CoA synthetase, Mco, and staphopain A in TSB, along with the elevated abundance of Mqo1 and Nuc in milk, reflects metabolic reprogramming that enhances energy production and oxidative stress responses to support bacterial survival. These proteomic shifts align with the enrichment of pathways related to lipid biosynthesis, energy metabolism, and nucleotide metabolism, indicating that *S. aureus* flexibly modulates its metabolic network to utilize available nutrients of each medium. Collectively, the divergent proteomic profiles observed in milk and TSB represent environment-specific adaptive responses to nutrient composition and metabolic demands. This interactive regulation highlights that the proteomic landscape of *S. aureus* in milk results from a dynamic bacterium–environment interplay, underscoring the pivotal role of environmental context in shaping bacterial metabolism, physiology, and pathogenic potential. 

## Conclusion

Dynamic changes in cell associated and secreted proteins of *S. aureus* cultured in both TSB and milk media were investigated using a proteomic approach. The results demonstrated that several cell associated proteins and secretory proteins, such as Hld, Hlb, and LukF-PV were increased in TSB and milk media across 3, 9 and 18 h. Notably, several virulence factors, including SarA and Sle1, were found to be present at higher levels in milk than in TSB medium, while Nuc was uniquely identified in milk. This highlighting the potential risk posed by *S. aureus* in milk and indicating its possible impact on food safety and consumer health. These results suggest that the nutrient composition of milk drives distinct metabolic and functional adaptations in *S. aureus*, promoting the preferential expression of stress response pathways and virulence factors. This study provides important insights into the metabolic reprogramming and functional adaptation of *S. aureus* under different growth conditions. Given that a specific isolate was used, future studies will expand the analytical framework to include a broader range of serotypes and antimicrobial resistance profiles, analyzed through proteomics, to obtain more robust and generalizable results. Overall, these findings enhance our understanding of the dynamic changes in the *S. aureus* secretome and have important implications for future preventive and control strategies, thereby enriching our understanding of the evolving cell associated protein and secreted protein profiles of *S. aureus*.

## Supplementary Information

Supplementary Material 1.

Supplementary Material 2.

## Data Availability

All data generated or analysed during this study are included in this published article [and its supplementary information files].

## Abbreviations

AgrA: Accessory gene regulator A
EsxA: Type VII secretion system extracellular protein A
FadA: Acetyl-CoA acyltransferase
FadD: Acyl-CoA synthetase
Hlb: Phospholipase C
HlgB: γ-hemolysin component B
Hld: Delta-hemolysin
KEGG: Kyoto Encyclopedia of Genes and Genomes
LFQ: Label-free quantification
LukF-PV: Panton-Valentine leukocidin F
Mco: Multicopper oxidase
Mqo1: Quinone oxidoreductase 1
Nuc: Thermonuclease
PCA: Principal component analyses
PepT: Peptidase T
Sle1: N-acetylmuramoyl-L-alanine amidase sle1
SarA: Transcriptional regulator sarA
SodM: Superoxide dismutase [Mn/Fe]
UreC: Urease subunit alpha

## References

[1] Tegegne H, Ejigu E, Woldegiorgis D, Mengistu A. Isolation and antimicrobial resistance patterns of Methicillin-resistant *Staphylococcus aureus* from raw cow’s milk in dairy farms of Wolaita Sodo Town, Southwest, Ethiopia. Food Sci Nutr. 2024;12(7):4735–44. 10.1002/fsn3.4121 39055220 10.1002/fsn3.4121 PMC11266931

[2] Perdomo A, Calle A. Assessment of microbial communities in a dairy farm from a food safety perspective. Int J Food Microbiol. 2024;423:110827. 10.1016/j.ijfoodmicro.2024.110827. 39043054 10.1016/j.ijfoodmicro.2024.110827

[3] Oliveira R, Pinho E, Almeida G, Azevedo NF, Almeida C. Prevalence and diversity of *Staphylococcus aureus* and Staphylococcal enterotoxins in raw milk from northern Portugal. Front Microbiol. 2022;13:846653. 10.3389/fmicb.2022.846653 35391724 10.3389/fmicb.2022.846653 PMC8981150

[4] Deddefo A, Mamo G, Asfaw M, Edao A, Hiko A, Fufa D, Jafer M, Sombo M, Amenu K. Occurrence, antimicrobial susceptibility, and resistance genes of *Staphylococcus aureus* in milk and milk products in the Arsi highlands of Ethiopia. BMC Microbiol. 2024;24(1):127. 10.1186/s12866-024-03288-3. 38627609 10.1186/s12866-024-03288-3 PMC11020821

[5] Abdelmegid S, Kelton D, Caswell J, Kirby G. Proteomic 2D-DIGE analysis of milk whey from dairy cows with *Staphylococcus aureus* mastitis reveals overexpression of host defense proteins. Microorganisms. 2020;8(12):1883. 10.3390/microorganisms8121883 33260718 10.3390/microorganisms8121883 PMC7760247

[6] Bentayeb L, Akkou M, Si-ahmed SZ, Titouche Y, Doumandji A, Megateli S. Impacts of subclinical mastitis on milk quality, clotting ability and microbial resistance of the causative *Staphylococci*. Large Anim Rev. 2023;29(3):105–11.

[7] González-Machado C, Capita R, Alonso-Calleja C. Methicillin-resistant *Staphylococcus aureus* (MRSA) in dairy products and bulk-tank milk (BTM). Antibiotics. 2024;13(7):588. 10.3390/antibiotics13070588. 39061270 10.3390/antibiotics13070588 PMC11273636

[8] Saeed MM. Influence of milk and raw milk as carriers in disease transmission between humans and animals. Eur Sci Methodical J. 2024;2(4):15–35.

[9] Kumar A, Patyal A. An insight on microbial flora of milk and milk products. The Microbiology, pathogenesis and zoonosis of milk borne diseases. Elsevier; 2024. pp. 69–94. 10.1016/B978-0-443-13805-8.00004-1.

[10] Gopikrishnan M, Haryini S. Emerging strategies and therapeutic innovations for combating drug resistance in *Staphylococcus aureus* strains: A comprehensive review. J Basic Microbiol. 2024;64(5):2300579. 10.1002/jobm.202300579. 10.1002/jobm.202300579 38308076

[11] Cheung GY, Bae JS, Otto M. Pathogenicity and virulence of *Staphylococcus aureus*. Virulence. 2021;12(1):547–69. 10.1080/21505594.2021.1878688. 33522395 10.1080/21505594.2021.1878688 PMC7872022

[12] Zhang Z, Chen Y, Li X, Wang X, Li H. Detection of antibiotic resistance, virulence gene, and drug resistance gene of *Staphylococcus aureus* isolates from bovine mastitis. Microbiol Spectr. 2022;10(4):e00471–00422. 10.1128/spectrum.00471-22. 35758746 10.1128/spectrum.00471-22 PMC9431281

[13] Liebeke M, Lalk M. *Staphylococcus aureus* metabolic response to changing environmental conditions–a metabolomics perspective. Int J Med Microbiol. 2014;304(3–4):222–9. 10.1016/j.ijmm.2013.11.017. 24439195 10.1016/j.ijmm.2013.11.017

[14] Dursun AD, Samet U, Yavuz O, Yurt MNZ, TAŞBAŞI BB, Acar EE, Özalp VC. Determination of the effect of glucose, sucrose and sodium chloride addition in different culture media on biofilm formation of methicillin resistant *Staphylococcus aureus*. Anatol Curr Med J. 2022;4(2):152–7. 10.38053/acmj.1037458.

[15] Poudel S, Tsunemoto H, Seif Y, Sastry AV, Szubin R, Xu S, Machado H, Olson CA, Anand A, Pogliano J. Revealing 29 sets of independently modulated genes in *Staphylococcus aureus*, their regulators, and role in key physiological response. Proc Natl Acad Sci. 2020;117(29):17228–39. . 10.1073/pnas.2008413117 32616573 10.1073/pnas.2008413117 PMC7382225

[16] Alreshidi M, Dunstan M, Macdonald RHM, Singh MK, Roberts VK. Analysis of cytoplasmic and secreted proteins of *Staphylococcus aureus* revealed adaptive metabolic homeostasis in response to changes in the environmental conditions representative of the human wound site. Microorganisms. 2020;8(7):1082. 10.3390/microorganisms8071082. 32698515 10.3390/microorganisms8071082 PMC7409162

[17] Becher D, Hempel K, Sievers S, Zühlke D, Pane-Farre J, Otto A, Fuchs S, Albrecht D, Bernhardt J, Engelmann S. A proteomic view of an important human pathogen–towards the quantification of the entire *Staphylococcus aureus* proteome. PLoS ONE. 2009;4(12):e8176. 10.1371/journal.pone.0008176. 19997597 10.1371/journal.pone.0008176 PMC2781549

[18] Foster TJ. Surface proteins of *Staphylococcus aureus*. Microbiol Spectr. 2019;7(4). 10.1128/microbiolspec.gpp3-0046-2018. 10.1128/microbiolspec.gpp3-0046-2018 PMC10957221 31267926

[19] Park K-H, Greenwood-Quaintance KE, Cunningham SA, Rajagopalan G, Chia N, Jeraldo PR, Mandrekar J, Patel RJM. Lack of correlation of virulence gene profiles of *Staphylococcus aureus* bacteremia isolates with mortality. Microb Pathog. 2019;133:103543. 10.1016/j.micpath.2019.103543. 31102653 10.1016/j.micpath.2019.103543

[20] Kusch H, Engelmann S. Secrets of the secretome in *Staphylococcus aureus*. Int J Med Microbiol. 2014;304(2):133–41. 10.1016/j.ijmm.2013.11.005. 24424242 10.1016/j.ijmm.2013.11.005

[21] Tam K, Torres VJ. *Staphylococcus aureus* secreted toxins and extracellular enzymes. Microbiol Spectr. 2019;7(2). 10.1128/microbiolspec.gpp1123-0039-2018. 10.1128/microbiolspec.gpp3-0039-2018 PMC6422052 30873936

[22] Addis MF, Pisanu S, Monistero V, Gazzola A, Penati M, Filipe J, Di Mauro S, Cremonesi P, Castiglioni B, Moroni P. Comparative secretome analysis of *Staphylococcus aureus* strains with different within-herd intramammary infection prevalence. Virulence. 2022;13(1):174–90. 10.1080/21505594.2021.2024014. 35030987 10.1080/21505594.2021.2024014 PMC8765078

[23] Qiu Y, Xu D, Xia X, Zhang K, Aadil RM, Batool Z, Wang J. Five major two components systems of *Staphylococcus aureus* for adaptation in diverse hostile environment. Microb Pathog. 2021;159:105119. 10.1016/j.micpath.2021.105119. 34339796 10.1016/j.micpath.2021.105119

[24] Khoon LY, Neela V. Secretome of *Staphylococcus aureus*. Afr J Microbiol Res. 2010;4(7):500–8. 10.1016/j.ijmm.2013.11.005.

[25] Abril G, Villa AG, Barros-Velázquez T, Cañas J, Sánchez-Pérez B, Calo-Mata A, Carrera P. *Staphylococcus aureus* exotoxins and their detection in the dairy industry and mastitis. Toxins. 2020;12(9):537. 10.3390/toxins12090537. 32825515 10.3390/toxins12090537 PMC7551672

[26] Schubert J, Podkowik M, Bystroń J, Bania J. Production of Staphylococcal enterotoxins D and R in milk and meat juice by *Staphylococcus aureus* strains. Foodborne Pathog Dis. 2017;14(4):223–30. 10.1089/fpd.2016.2210. 28072918 10.1089/fpd.2016.2210

[27] Alreshidi M, Dunstan M, Macdonald RHM, Singh MK, Roberts VK. Analysis of cytoplasmic and secreted proteins of *Staphylococcus aureus* revealed adaptive metabolic homeostasis in response to changes in the environmental conditions representative of the human wound site. Microorganisms. 2020;8(7). 10.3390/microorganisms8071082. 10.3390/microorganisms8071082 PMC7409162 32698515

[28] Tyanova S, Temu T, Cox J. The MaxQuant computational platform for mass spectrometry-based shotgun proteomics. Nat Protoc. 2016;11(12):2301–19. 10.1038/nprot.2016.136. 27809316 10.1038/nprot.2016.136

[29] Kiedrowski MR, Paharik AE, Ackermann LW, Shelton AU, Singh SB, Starner TD, Horswill AR. Development of an in vitro colonization model to investigate *Staphylococcus aureus* interactions with airway epithelia. Cell Microbiol. 2016;18(5):720–32. 10.1111/cmi.12543. 26566259 10.1111/cmi.12543 PMC4840028

[30] Clark DP, Cronan JE. Two-carbon compounds and fatty acids as carbon sources. EcoSal Plus. 2005;1(2):1124. 10.1128/ecosalplus.3.4.4. 10.1128/ecosalplus.3.4.4 26443509

[31] Cintolesi A, Rodríguez-Moyá M, Gonzalez R. Fatty acid oxidation: systems analysis and applications. Wiley Interdisciplinary Reviews: Syst Biology Med. 2013;5(5):575–85. 10.1002/wsbm.1226. 10.1002/wsbm.1226 23661533

[32] Zapotoczna M, Riboldi Gustavo P, Moustafa Ahmed M, Dickson E, Narechania A, Morrissey Julie A, Planet Paul J, Holden Matthew TG, Waldron Kevin J, Geoghegan Joan A. Mobile-Genetic-Element-Encoded hypertolerance to copper protects *Staphylococcus aureus* from killing by host phagocytes. mBio. 2018;9(5):101128. 10.1128/mbio.00550-18. /mbio.00550 – 00518. 10.1128/mBio.00550-18 PMC6191537 30327441

[33] Gaupp R, Ledala N, Somerville GA. Staphylococcal response to oxidative stress. Front Cell Infect Microbiol. 2012;2:33. 10.3389/fcimb.2012.00033. 22919625 10.3389/fcimb.2012.00033 PMC3417528

[34] Kaur K, Sharma A, Capalash N, Sharma P. Multicopper oxidases: biocatalysts in microbial pathogenesis and stress management. Microbiol Res. 2019;222:1–13. 10.1016/j.micres.2019.02.007. 30928025 10.1016/j.micres.2019.02.007

[35] Sharma KK, Singh D, Mohite SV, Williamson PR, Kennedy JF. Metal manipulators and regulators in human pathogens: A comprehensive review on microbial redox copper metalloenzymes multicopper oxidases and superoxide dismutases. Int J Biol Macromol. 2023;233:123534. 10.1016/j.ijbiomac.2023.123534. 36740121 10.1016/j.ijbiomac.2023.123534

[36] Gabryszewski SJ, Wong Fok Lung T, Annavajhala MK, Tomlinson KL, Riquelme SA, Khan IN, Noguera LP, Wickersham M, Zhao A, Mulenos AM. Metabolic adaptation in methicillin-resistant *Staphylococcus aureus* pneumonia. Am J Respir Cell Mol Biol. 2019;61(2):185–97. 10.1165/rcmb.2018-0389OC. 30742488 10.1165/rcmb.2018-0389OC PMC6670030

[37] Hartuti ED, Inaoka DK, Komatsuya K, Miyazaki Y, Miller RJ, Xinying W, Sadikin M, Prabandari EE, Waluyo D, Kuroda M. Biochemical studies of membrane bound plasmodium falciparum mitochondrial L-malate: Quinone oxidoreductase, a potential drug target. Biochim Et Biophys Acta (BBA)-Bioenergetics. 2018;1859(3):191–200. 10.1016/j.bbabio.2017.12.004. 29269266 10.1016/j.bbabio.2017.12.004

[38] Liebeke M, Dörries K, Zühlke D, Bernhardt J, Fuchs S, Pane-Farre J, Engelmann S, Völker U, Bode R, Dandekar T. A metabolomics and proteomics study of the adaptation of *Staphylococcus aureus* to glucose starvation. Mol Biosyst. 2011;7(4):1241–53. 10.1039/c0mb00315h. 21327190 10.1039/c0mb00315h

[39] Zhou C, Bhinderwala F, Lehman MK, Thomas VC, Chaudhari SS, Yamada KJ, Foster KW, Powers R, Kielian T, Fey PD. Urease is an essential component of the acid response network of *Staphylococcus aureus* and is required for a persistent murine kidney infection. PLoS Pathog. 2019;15(1):e1007538. 10.1371/journal.ppat.1007538. 30608981 10.1371/journal.ppat.1007538 PMC6343930

[40] Chaffin DO, Taylor D, Skerrett SJ, Rubens CE. Changes in the *Staphylococcus aureus* transcriptome during early adaptation to the lung. PLoS ONE. 2012. 10.1371/journal.pone.0041329. 22876285 10.1371/journal.pone.0041329 PMC3410880

[41] Patel K, Godden SM, Royster EE, Crooker BA, Johnson TJ, Smith EA, Sreevatsan S. Prevalence, antibiotic resistance, virulence and genetic diversity of *Staphylococcus aureus* isolated from bulk tank milk samples of US dairy herds. BMC Genomics. 2021;22(1):367. 10.1186/s12864-021-07603-4. 34016049 10.1186/s12864-021-07603-4 PMC8135151

[42] Eidaroos NH, Algammal AM, Mohamaden WI, Alenzi AM, Alghamdi S, Kabrah A, El-Mahallawy HS, Eid HM, Algwad AA, Asfor SA. Virulence traits, Agr typing, multidrug resistance patterns, and biofilm ability of MDR *Staphylococcus aureus* recovered from clinical and subclinical mastitis in dairy cows. BMC Microbiol. 2025;25(1):155. 10.1186/s12866-025-03870-3. 40102767 10.1186/s12866-025-03870-3 PMC11921537

[43] Vandenesch F, Lina G, Henry T. *Staphylococcus aureus* hemolysins, bi-component leukocidins, and cytolytic peptides: a redundant arsenal of membrane-damaging virulence factors? Front Cell Infect Microbiol. 2012;2:12. 10.3389/fcimb.2012.00012. 22919604 10.3389/fcimb.2012.00012 PMC3417661

[44] Divyakolu S, Chikkala R, Ratnakar KS, Sritharan V. Hemolysins of *Staphylococcus aureus*—an update on their biology, role in pathogenesis and as targets for anti-virulence therapy. Adv Infect Dis. 2019;9(2):80–104. 10.4236/aid.2019.92007.

[45] Yu F, Liu Y, Xu Y, Shang Y, Lou D, Qin Z, Parsons C, Zhou W, Huang X, Li Y. Expression of *Panton-Valentine leukocidin* mRNA among *Staphylococcus aureus* isolates associates with specific clinical presentations. PLoS ONE. 2013;8(12):e83368. . 10.1371/journal.pone.0083368 24349495 10.1371/journal.pone.0083368 PMC3861483

[46] Vrieling M, Koymans K, Heesterbeek D, Aerts P, Rutten V, De Haas C, Van Kessel K, Koets A, Nijland R, van Strijp J. Bovine *Staphylococcus aureus* secretes the leukocidin LukMF′ to kill migrating neutrophils through CCR1. MBio. 2015;6(3):e00335–00315. 10.1128/mbio.00335-15 26045537 10.1128/mBio.00335-15 PMC4462618

[47] Spaan AN, van Strijp JA, Torres VJ. Leukocidins: Staphylococcal bi-component pore-forming toxins find their receptors. Nat Rev Microbiol. 2017;15(7):435–47. 10.1038/nrmicro.2017.27. 28420883 10.1038/nrmicro.2017.27 PMC5621924

[48] Kawada-Matsuo M, Yoshida Y, Nakamura N, Komatsuzawa H. Role of two-component systems in the resistance of *Staphylococcus aureus* to antibacterial agents. Virulence. 2011;2(5):427–30. 10.4161/viru.2.5.17711. 21921684 10.4161/viru.2.5.17711

[49] Treffon J, Block D, Moche M, Reiss S, Fuchs S, Engelmann S, Becher D, Langhanki L, Mellmann A, Peters G. Adaptation of *Staphylococcus aureus* to airway environments in patients with cystic fibrosis by upregulation of superoxide dismutase M and iron-scavenging proteins. J Infect Dis. 2018;217(9):1453–61. 10.1093/infdis/jiy012. 29325044 10.1093/infdis/jiy012

[50] Valderas MW, Gatson JW, Wreyford N, Hart ME. The superoxide dismutase gene *SodM* is unique to *Staphylococcus aureus*: absence of *SodM* in coagulase-negative *Staphylococci*. J Bacteriol. 2002;184(9):2465–72. . 10.1128/jb.184.9.2465-2472.2002 11948161 10.1128/JB.184.9.2465-2472.2002 PMC134988

[51] Hayyan M, Hashim MA, AlNashef IM. Superoxide ion: generation and chemical implications. Chem Rev. 2016;116(5):3029–85. 10.1021/acs.chemrev.5b00407. 26875845 10.1021/acs.chemrev.5b00407

[52] Garcia YM, Barwinska-Sendra A, Tarrant E, Skaar EP, Waldron KJ, Kehl-Fie TE. A superoxide dismutase capable of functioning with iron or manganese promotes the resistance of *Staphylococcus aureus* to calprotectin and nutritional immunity. PLoS Pathog. 2017;13(1):e1006125. 10.1371/journal.ppat.1006125. 28103306 10.1371/journal.ppat.1006125 PMC5245786

[53] Grażyna C, Hanna C, Adam A, Magdalena BM. Natural antioxidants in milk and dairy products. Int J Dairy Technol. 2017;70(2):165–78. 10.1111/1471-0307.12359.

[54] Torres NJ, Rizzo DN, Reinberg MA, Jobson M-E, Totzke BC, Jackson JK, Yu W, Shaw LN. The identification of two M20B family peptidases required for full virulence in *Staphylococcus aureus*. Front Cell Infect Microbiol. 2023;13:1176769. 10.1016/j.cmi.2016.06.020. 37538308 10.3389/fcimb.2023.1176769 PMC10394242

[55] Zheng X. The role of the cell envelope in *Staphylococcus aureus* protein secretion and resistance to host antimicrobial proteins. New York University; 2022.

[56] Herzog S, Dach F, De Buhr N, Niemann S, Schlagowski J, Chaves-Moreno D, Neumann C, Goretzko J, Schwierzeck V, Mellmann A. High nuclease activity of long persisting *Staphylococcus aureus* isolates within the airways of cystic fibrosis patients protects against NET-mediated killing. Front Immunol. 2019;10:2552. 10.3389/fimmu.2019.02552. 31772562 10.3389/fimmu.2019.02552 PMC6849659

[57] Hu Y, Meng J, Shi C, Hervin K, Fratamico PM, Shi X. Characterization and comparative analysis of a second thermonuclease from *Staphylococcus aureus*. Microbiol Res. 2013;168(3):174–82. 10.1016/j.micres.2012.09.003. 23295145 10.1016/j.micres.2012.09.003

[58] Sharma P, Garg N, Sharma A, Capalash N, Singh R. Nucleases of bacterial pathogens as virulence factors, therapeutic targets and diagnostic markers. Int J Med Microbiol. 2019;309(8):151354. 10.1016/j.ijmm.2019.151354. 31495663 10.1016/j.ijmm.2019.151354

[59] Ziegler I, Cajander S, Rasmussen G, Ennefors T, Mölling P, Strålin K. High Nuc DNA load in whole blood is associated with sepsis, mortality and immune dysregulation in *Staphylococcus aureus* bacteraemia. Infect Dis. 2019;51(3):216–26. 10.1080/23744235.2018.1562205. 10.1080/23744235.2018.1562205 30676833

