# Supplementary material for: Insights into the dynamic cell associated and secreted proteome of Staphylococcus aureus cultured in TSB and milk media: a proteomic analysis
Source: BMC Microbiol. 2025 Dec 27;26:93. doi: 10.1186/s12866-025-04587-z (PMC12882269; doi:10.1186/s12866-025-04587-z)
Supplement: Supplementary file 2 — Supplementary Material 2. [file 12866_2025_4587_MOESM2_ESM.docx]

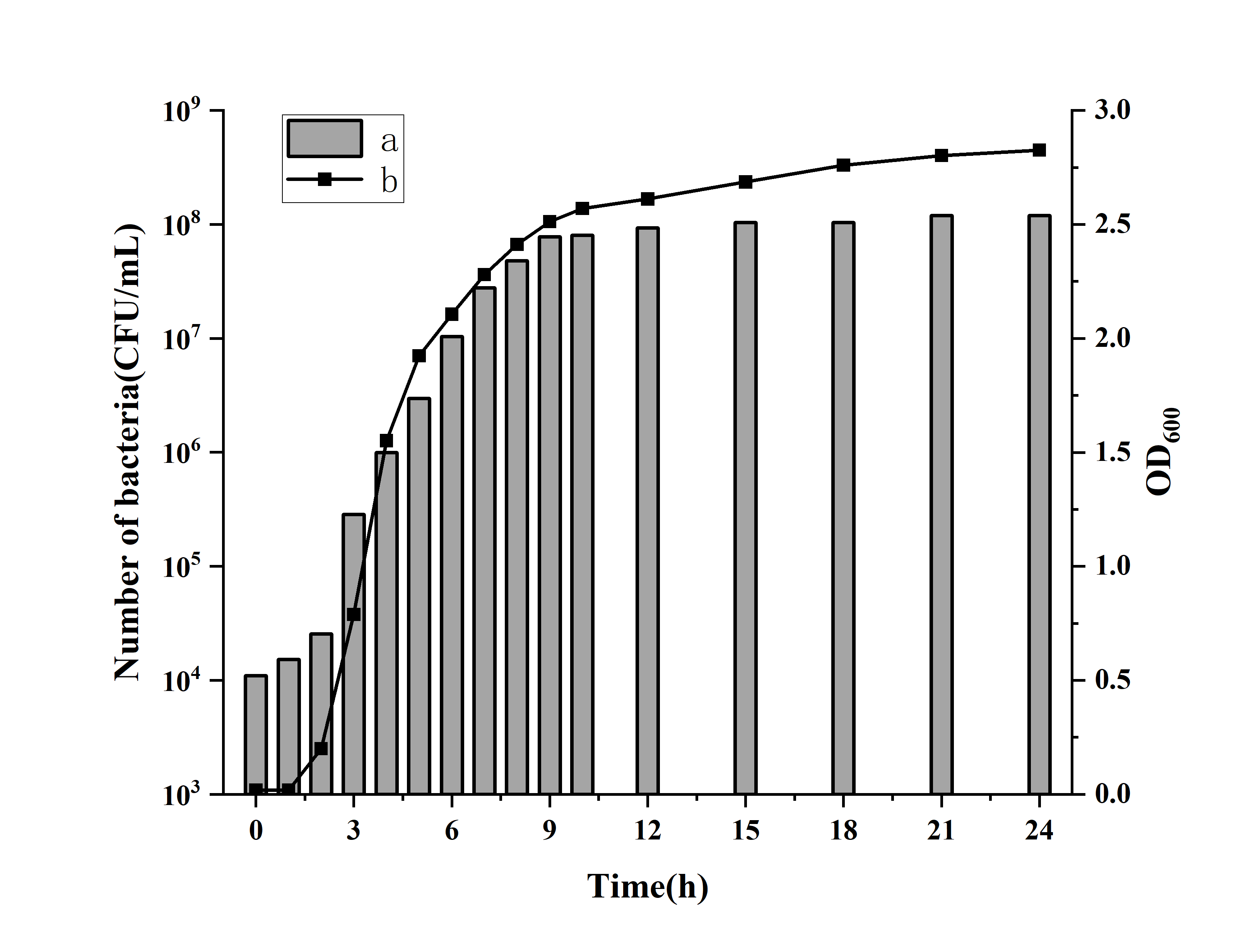


Fig. S1: Bacterial growth monitored in TSB medium via optical density 600 and in milk by colony-forming units.

**(a)**

**(b)**


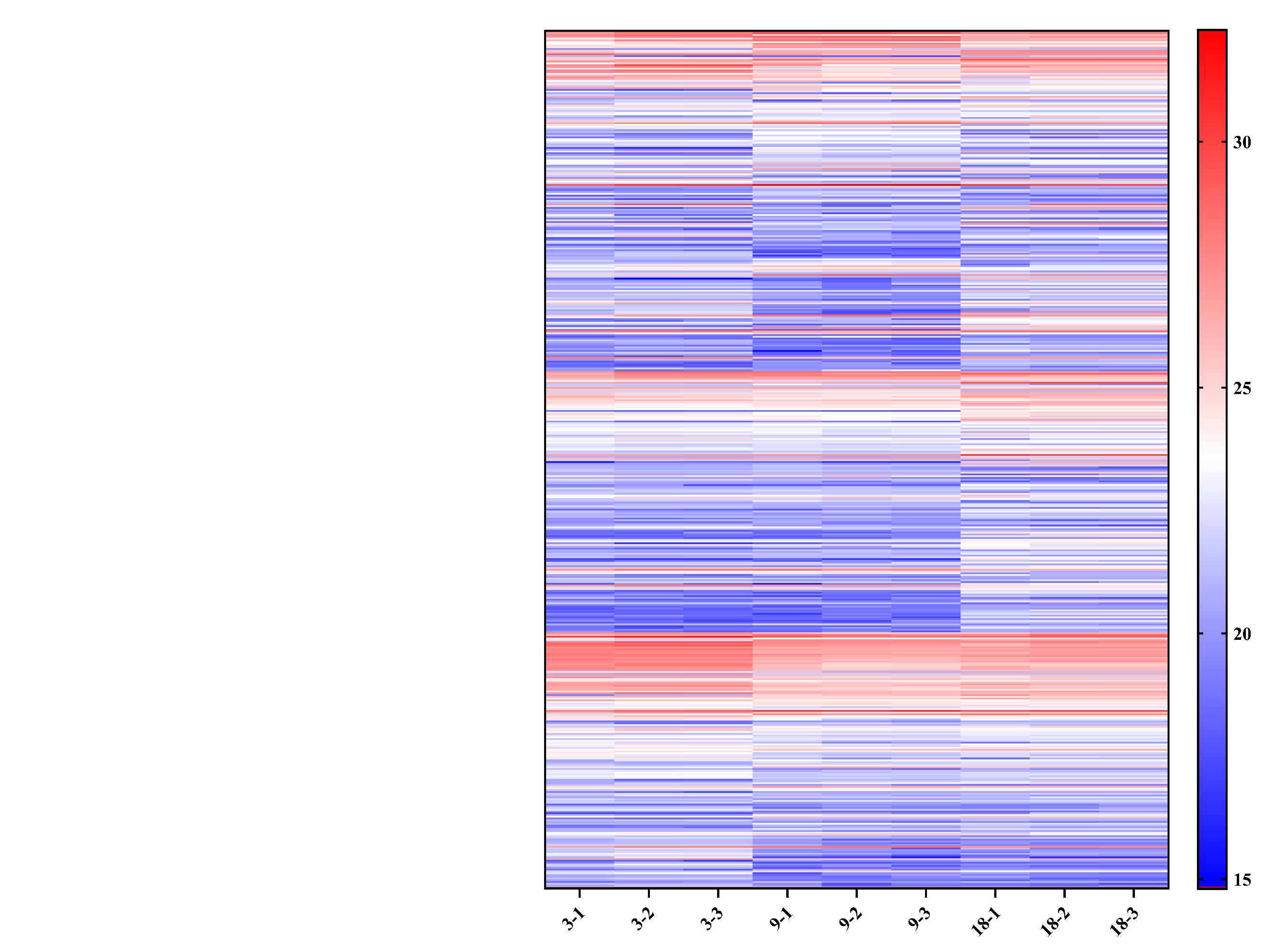

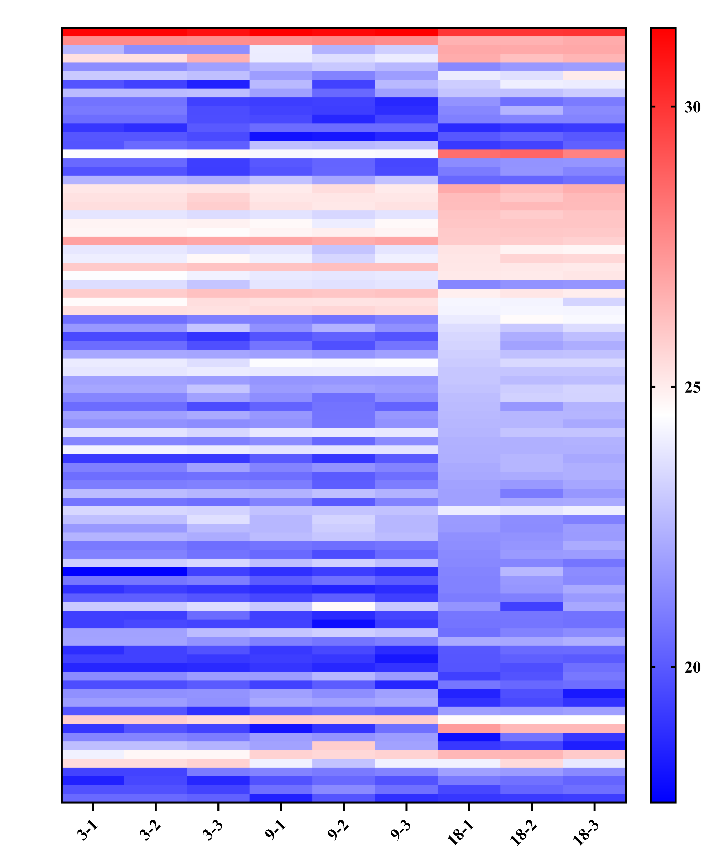


Figures S2 Clustered map of differential cell associated proteins of *S. aureus* cultured in TSB (a) and milk media (b).


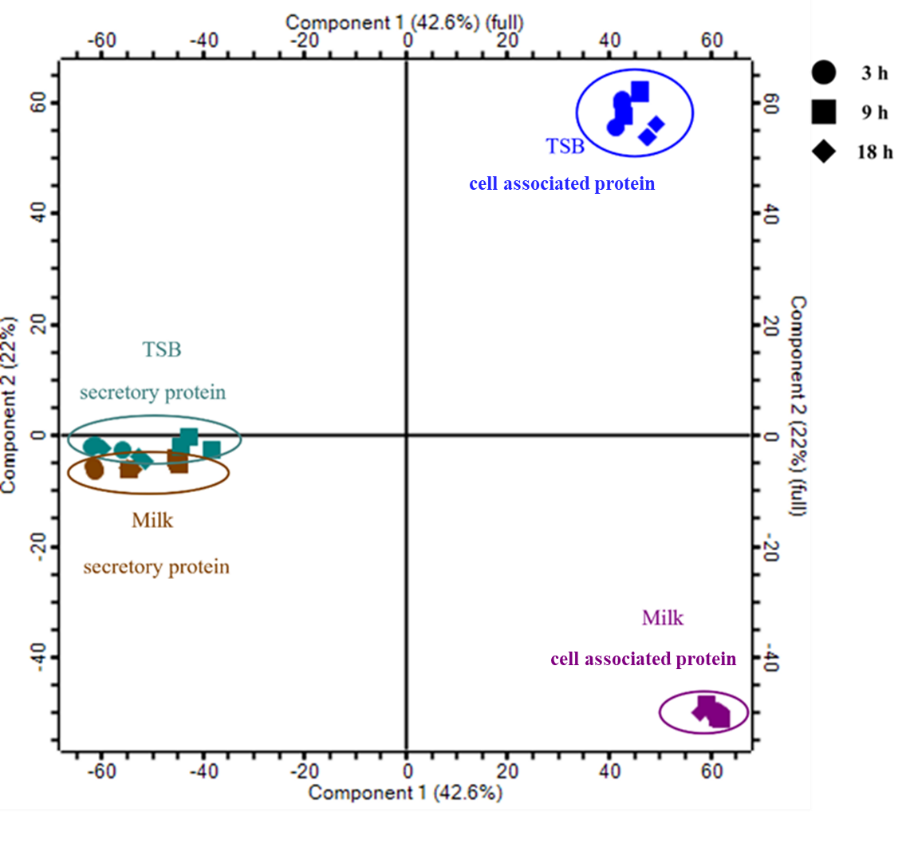
Fig. S3 Principal component analysis of cell-associated and secreted proteins of *S. aureus* cultured in TSB and milk media using a DDA-based proteomic approach. Circles, squares, and diamonds represent samples collected after 3, 9, and 18 h of incubation, respectively.

**(a)**

**(b)**


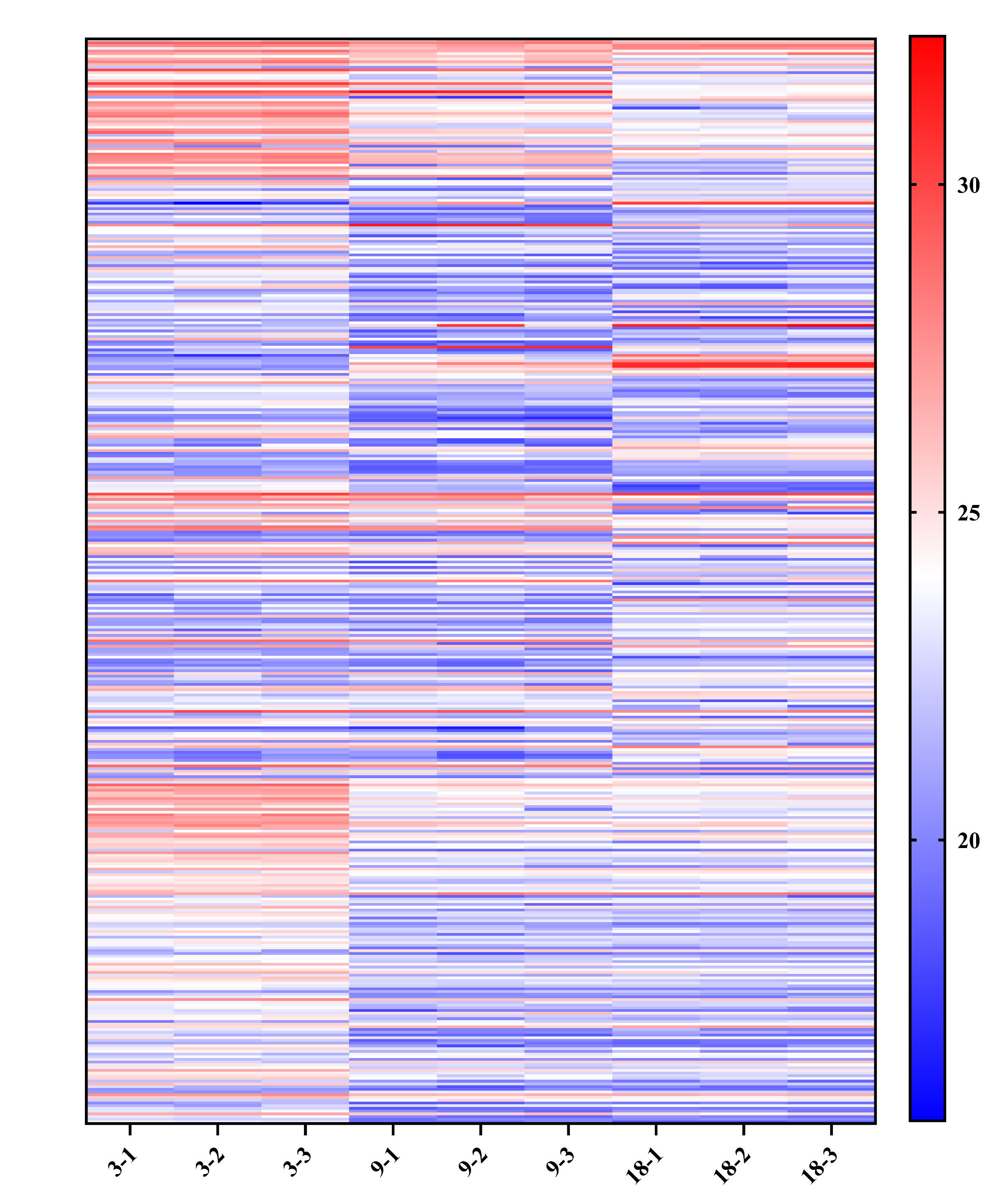

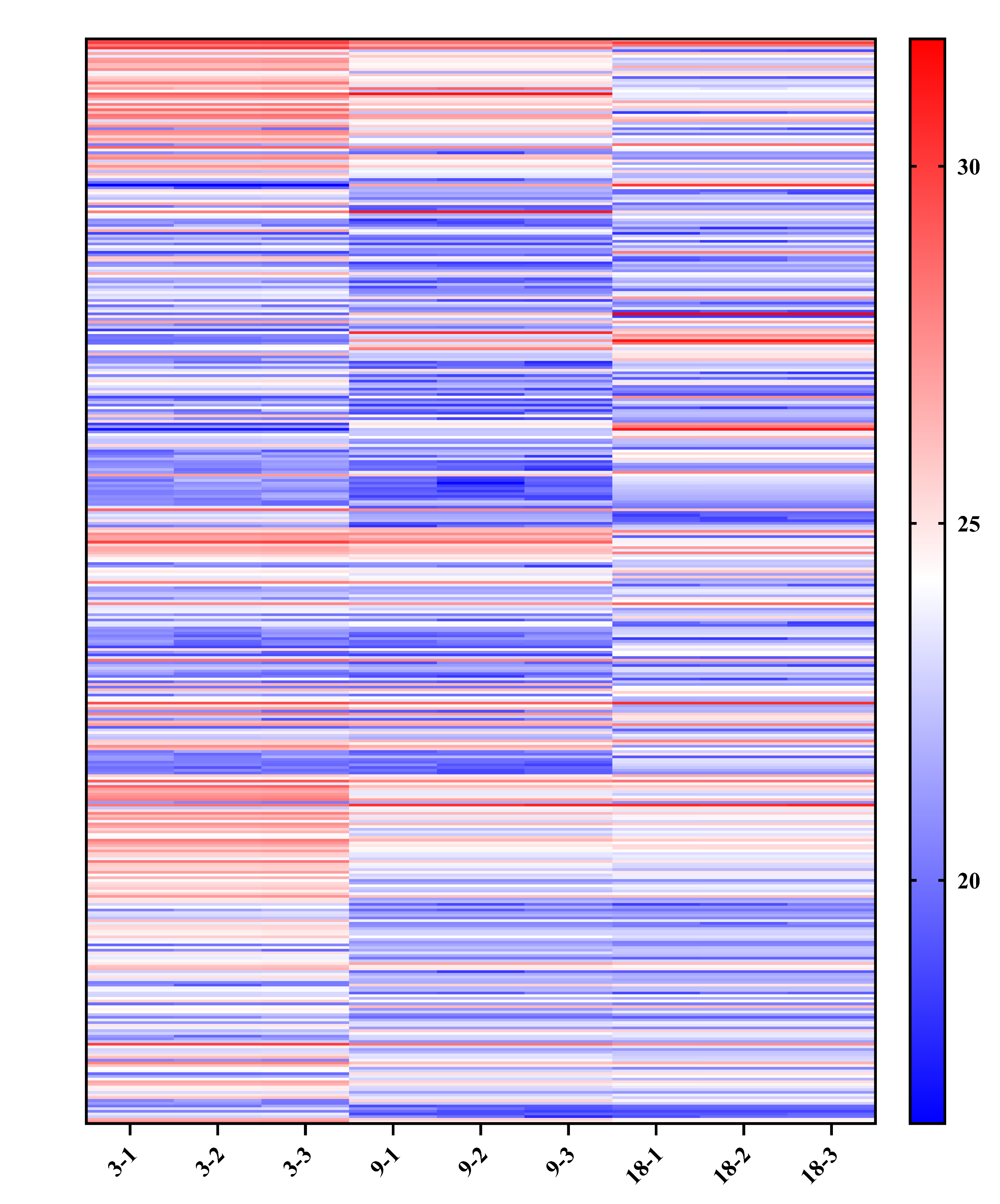


Figures S4 Clustered map of differential secreted proteins of *S. aureus* cultured in TSB (a) and milk media (b).

Table S1. Primers and sequences for RT-qPCR experiments

| **Gene** | **Sequence 5′ to 3′** | **References** |
| --- | --- | --- |
| *gyrB* | F: ACCATAATGTAGCAGCCTCTTG | [1] |
|  | R: GGCATGGGTTAGAAATAGATGGA |  |
| *Mco* | F: ACCATAATGTAGCAGCCTCTTG | [1] |
|  | R: GGCATGGGTTAGAAATAGATGGA |  |
| *mqo1* | F: ATGCTATCGTGATGAATTCAAAGG | [2] |
|  | R: TTATTTAACTTAAATACTTAGTTAC |  |
| *agrA* | F: CGAATGCCTACACATCAAGGTA | [3] |
|  | R: TCACCACACCTTTTGTCGTATC |  |
| *hld* | F: GAGTTGTTTAATTTTAAG | [4] |
|  | R: TTTTAGTGAATTTGT |  |
| *ureC* | F:GTAGGATCTATCGAGCCGGG | [5] |
|  | R:CATCGCCATTTACGGCAGAG |  |

Table S2. KEGG pathway analysis of differential cell associated proteins of *S. aureus* cultured in TSB and milk media

| **Terms** | **count** | **Hits** | **P-Value** | **Enrich Factor** |
| --- | --- | --- | --- | --- |
| **TSB medium** | | | | |
| Carbohydrate metabolism | 70 | 8414 | 1.73E-03 | 1.42 |
| Amino acid metabolism | 65 | 6860 | 8.42E-05 | 1.61 |
| Genetic Information Processing | 61 | 8089 | 2.49E-02 | 1.28 |
| Translation | 47 | 3431 | 8.23E-08 | 2.33 |
| Ribosome | 38 | 2049 | 6.72E-10 | 3.16 |
| Energy metabolism | 38 | 4204 | 5.87E-03 | 1.54 |
| Nucleotide metabolism | 27 | 2933 | 1.52E-02 | 1.57 |
| Pyruvate metabolism | 23 | 2253 | 7.97E-03 | 1.74 |
| Glycolysis / Gluconeogenesis | 20 | 1983 | 1.44E-02 | 1.72 |
| Purine metabolism | 19 | 2040 | 3.41E-02 | 1.59 |
| Chaperones and folding catalysts | 15 | 1070 | 1.90E-03 | 2.39 |
| Citrate cycle (TCA cycle) | 15 | 1097 | 2.41E-03 | 2.33 |
| Xenobiotics biodegradation and metabolism | 15 | 1226 | 6.58E-03 | 2.08 |
| Glyoxylate and dicarboxylate metabolism | 15 | 1371 | 1.68E-02 | 1.86 |
| Arginine biosynthesis | 14 | 873 | 7.69E-04 | 2.73 |
| Carbon fixation pathways in prokaryotes | 14 | 1241 | 1.62E-02 | 1.92 |
| Pyrimidine metabolism | 13 | 1288 | 4.28E-02 | 1.72 |
| Butanoate metabolism | 12 | 804 | 3.19E-03 | 2.54 |
| Alanine, aspartate and glutamate metabolism | 11 | 901 | 1.87E-02 | 2.08 |
| Arginine and proline metabolism | 10 | 575 | 2.36E-03 | 2.96 |
| Nitrogen metabolism | 8 | 672 | 4.65E-02 | 2.03 |
| **milk medium** |  |  |  |  |
| Carbohydrate metabolism | 136 | 8414 | 3.38E-04 | 1.33 |
| Translation | 78 | 3431 | 1.18E-07 | 1.87 |
| Energy metabolism | 77 | 4204 | 2.54E-04 | 1.51 |
| Ribosome | 53 | 2049 | 3.29E-07 | 2.13 |
| Transfer RNA biogenesis | 50 | 3046 | 2.07E-02 | 1.35 |
| Pyruvate metabolism | 43 | 2253 | 2.78E-03 | 1.57 |
| Exosome | 41 | 1807 | 1.29E-04 | 1.87 |
| Glycolysis / Gluconeogenesis | 40 | 1983 | 1.48E-03 | 1.66 |
| Ribosome biogenesis | 37 | 2150 | 2.32E-02 | 1.42 |
| Mitochondrial biogenesis | 31 | 1538 | 4.82E-03 | 1.66 |
| Chaperones and folding catalysts | 26 | 1070 | 8.04E-04 | 2.00 |
| Glyoxylate and dicarboxylate metabolism | 25 | 1371 | 3.10E-02 | 1.50 |
| Aminoacyl-tRNA biosynthesis | 25 | 1382 | 3.36E-02 | 1.49 |
| Pentose phosphate pathway | 22 | 1027 | 8.33E-03 | 1.76 |
| Propanoate metabolism | 18 | 832 | 1.45E-02 | 1.78 |
| Oxidative phosphorylation | 18 | 933 | 3.88E-02 | 1.59 |
| Arginine biosynthesis | 17 | 873 | 4.06E-02 | 1.60 |
| Carbon fixation in photosynthetic organisms | 15 | 598 | 7.22E-03 | 2.07 |
| Translation factors | 14 | 666 | 3.52E-02 | 1.73 |
| Membrane trafficking | 11 | 300 | 1.25E-03 | 3.02 |
| Salmonella infection | 7 | 135 | 1.38E-03 | 4.27 |
| Organismal Systems | 6 | 156 | 1.24E-02 | 3.17 |

Table S3. KEGG pathway analysis of differential secreted proteins of *S. aureus* cultured in TSB and milk media

| **Term** | **Count** | **Hits** | **p-value** | **Enrich Factor** |  |
| --- | --- | --- | --- | --- | --- |
| **TSB medium** | | | | | |
| Carbohydrate metabolism | 59 | 8414 | 4.17E-03 | 1.41 |  |
| Translation | 55 | 3431 | 2.23E-14 | 3.23 |  |
| Amino acid metabolism | 44 | 6860 | 4.85E-02 | 1.29 |  |
| Energy metabolism | 40 | 4204 | 7.15E-05 | 1.91 |  |
| Ribosome | 35 | 2049 | 3.38E-10 | 3.44 |  |
| Infectious disease: bacterial | 26 | 2563 | 5.44E-04 | 2.04 |  |
| Pyruvate metabolism | 24 | 2253 | 4.50E-04 | 2.14 |  |
| Protein families: metabolism | 53 | 8221 | 2.94E-02 | 1.30 |  |
| Human Diseases | 29 | 3724 | 1.20E-02 | 1.57 |  |
| Mitochondrial biogenesis | 23 | 1538 | 3.72E-06 | 3.01 |  |
| Exosome | 23 | 1807 | 4.65E-05 | 2.56 |  |
| Glycolysis / Gluconeogenesis | 23 | 1983 | 1.81E-04 | 2.33 |  |
| Aminoacyl-tRNA biosynthesis | 20 | 1382 | 2.54E-05 | 2.91 |  |
| Amino acid related enzymes | 19 | 1681 | 8.82E-04 | 2.27 |  |
| Staphylococcus aureus infection | 19 | 1994 | 5.72E-03 | 1.92 |  |
| Citrate cycle (TCA cycle) | 17 | 1097 | 4.47E-05 | 3.12 |  |
| Carbon fixation pathways in prokaryotes | 16 | 1241 | 5.73E-04 | 2.59 |  |
| Chaperones and folding catalysts | 15 | 1070 | 3.60E-04 | 2.82 |  |
| Glyoxylate and dicarboxylate metabolism | 14 | 1371 | 9.49E-03 | 2.05 |  |
| Pyrimidine metabolism | 13 | 1288 | 1.33E-02 | 2.03 |  |
| Propanoate metabolism | 12 | 832 | 1.07E-03 | 2.90 |  |
| Glycerolipid metabolism | 12 | 840 | 1.17E-03 | 2.87 |  |
| Arginine biosynthesis | 12 | 873 | 1.61E-03 | 2.77 |  |
| Alanine, aspartate and glutamate metabolism | 10 | 901 | 1.57E-02 | 2.23 |  |
| Bacterial toxins | 9 | 675 | 7.19E-03 | 2.68 |  |
| Transcription machinery | 9 | 781 | 1.72E-02 | 2.32 |  |
| Arginine and proline metabolism | 8 | 575 | 8.64E-03 | 2.80 |  |
| Translation factors | 8 | 666 | 1.93E-02 | 2.42 |  |
| Fatty acid degradation | 8 | 733 | 3.15E-02 | 2.20 |  |
| Methane metabolism | 8 | 793 | 4.63E-02 | 2.03 |  |
| Lysine degradation | 7 | 534 | 1.82E-02 | 2.64 |  |
| Valine, leucine and isoleucine degradation | 7 | 595 | 3.04E-02 | 2.37 |  |
| Tryptophan metabolism | 7 | 607 | 3.33E-02 | 2.32 |  |
| Lipid biosynthesis proteins | 7 | 645 | 4.38E-02 | 2.18 |  |
| Nicotinate and nicotinamide metabolism | 6 | 487 | 3.60E-02 | 2.48 |  |
| Membrane trafficking | 5 | 300 | 1.76E-02 | 3.35 |  |
| RNA polymerase | 5 | 306 | 1.90E-02 | 3.29 |  |
| Transcription | 5 | 306 | 1.90E-02 | 3.29 |  |
| **milk medium** | | | | |  |
| Carbohydrate metabolism | 68 | 8414 | 7.00E-05 | 1.59 |  |
| Translation | 48 | 3431 | 2.59E-10 | 2.76 |  |
| Amino acid metabolism | 48 | 6860 | 1.42E-02 | 1.38 |  |
| Energy metabolism | 40 | 4204 | 1.09E-04 | 1.88 |  |
| Ribosome | 31 | 2049 | 8.80E-08 | 2.99 |  |
| Lipid metabolism | 27 | 2747 | 9.18E-04 | 1.94 |  |
| Glycolysis / Gluconeogenesis | 26 | 1983 | 1.26E-05 | 2.59 |  |
| Pyruvate metabolism | 24 | 2253 | 5.94E-04 | 2.10 |  |
| Exosome | 23 | 1807 | 6.26E-05 | 2.51 |  |
| Nucleotide metabolism | 23 | 2933 | 2.70E-02 | 1.55 |  |
| Infectious disease: bacterial | 22 | 2563 | 1.22E-02 | 1.69 |  |
| Citrate cycle (TCA cycle) | 18 | 1097 | 1.65E-05 | 3.24 |  |
| Mitochondrial biogenesis | 18 | 1538 | 1.01E-03 | 2.31 |  |
| Amino acid related enzymes | 18 | 1681 | 2.65E-03 | 2.11 |  |
| Aminoacyl-tRNA biosynthesis | 17 | 1382 | 8.13E-04 | 2.43 |  |
| Chaperones and folding catalysts | 16 | 1070 | 1.40E-04 | 2.95 |  |
| Xenobiotics biodegradation and metabolism | 16 | 1226 | 6.21E-04 | 2.57 |  |
| Glyoxylate and dicarboxylate metabolism | 16 | 1371 | 1.95E-03 | 2.30 |  |
| Metabolism of other amino acids | 15 | 1762 | 3.69E-02 | 1.68 |  |
| Carbon fixation pathways in prokaryotes | 14 | 1241 | 4.88E-03 | 2.23 |  |
| Glycerolipid metabolism | 13 | 840 | 4.23E-04 | 3.05 |  |
| Arginine biosynthesis | 12 | 873 | 1.89E-03 | 2.71 |  |
| Fatty acid degradation | 11 | 733 | 1.47E-03 | 2.96 |  |
| Transcription machinery | 10 | 781 | 7.10E-03 | 2.53 |  |
| Alanine, aspartate and glutamate metabolism | 10 | 901 | 1.77E-02 | 2.19 |  |
| Pentose phosphate pathway | 10 | 1027 | 3.83E-02 | 1.92 |  |
| Lysine degradation | 9 | 534 | 1.79E-03 | 3.33 |  |
| Valine, leucine and isoleucine degradation | 9 | 595 | 3.66E-03 | 2.98 |  |
| Tryptophan metabolism | 9 | 607 | 4.16E-03 | 2.93 |  |
| Propanoate metabolism | 9 | 832 | 2.74E-02 | 2.13 |  |
| Arginine and proline metabolism | 8 | 575 | 9.65E-03 | 2.75 |  |
| Benzoate degradation | 6 | 450 | 2.81E-02 | 2.63 |  |
| Membrane trafficking | 5 | 300 | 1.90E-02 | 3.29 |  |

1. Zapotoczna, M., et al., *Mobile-Genetic-Element-Encoded Hypertolerance to Copper Protects Staphylococcus aureus from Killing by Host Phagocytes.* mBio, 2018. **9**(5): p. 10.1128/mbio.00550-18.<http://https://doi.org/10.1128/mbio.00550-18>.

2. Gabryszewski, S.J., et al., *Metabolic adaptation in methicillin-resistant Staphylococcus aureus pneumonia.* American Journal of Respiratory Cell and Molecular Biology, 2019. **61**(2): p. 185-197.<http://https://doi.org/10.1165/rcmb.2018-0389OC>.

3. Autret, N., et al., *Identification of the agr locus of Listeria monocytogenes: role in bacterial virulence.* Infection and immunity, 2003. **71**(8): p. 4463-4471.<http://https://doi.org/10.1128/iai.71.8.4463-4471.2003>.

4. Zhang, H., et al., *Identification and characterization of Staphylococcus aureus strains with an incomplete hemolytic phenotype.* Frontiers in cellular and infection microbiology, 2016. **6**: p. 146.<http://https://doi.org/10.3389/fcimb.2016.00146>.

5. Ghosh, P., et al., *Transcriptional reprogramming under vancomycin pressure in Staphylococcus aureus.* 2023.<http://10.56042/ijeb.v61i07.2211>
